# ARMOUR – A Rice miRNA: mRNA Interaction Resource

**DOI:** 10.3389/fpls.2018.00602

**Published:** 2018-05-08

**Authors:** Neeti Sanan-Mishra, Anita Tripathi, Kavita Goswami, Rohit N. Shukla, Madavan Vasudevan, Hitesh Goswami

**Affiliations:** ^1^Plant RNAi Biology Group, International Centre for Genetic Engineering and Biotechnology, New Delhi, India; ^2^Genome Informatics Research Group, Bionivid Technology Private Limited, Bengaluru, India

**Keywords:** miRNA, database, target, gene annotation, pathway, expression

## Abstract

ARMOUR was developed as A Rice miRNA:mRNA interaction resource. This informative and interactive database includes the experimentally validated expression profiles of miRNAs under different developmental and abiotic stress conditions across seven Indian rice cultivars. This comprehensive database covers 689 known and 1664 predicted novel miRNAs and their expression profiles in more than 38 different tissues or conditions along with their predicted/known target transcripts. The understanding of miRNA:mRNA interactome in regulation of functional cellular machinery is supported by the sequence information of the mature and hairpin structures. ARMOUR provides flexibility to users in querying the database using multiple ways like known gene identifiers, gene ontology identifiers, KEGG identifiers and also allows on the fly fold change analysis and sequence search query with inbuilt BLAST algorithm. ARMOUR database provides a cohesive platform for novel and mature miRNAs and their expression in different experimental conditions and allows searching for their interacting mRNA targets, GO annotation and their involvement in various biological pathways. The ARMOUR database includes a provision for adding more experimental data from users, with an aim to develop it as a platform for sharing and comparing experimental data contributed by research groups working on rice.

## Introduction

MicroRNAs are a class of non-coding RNAs that are transcribed from different loci in the genome and play a vital role in the regulation of gene expression at the transcriptional and post-transcriptional levels (Carthew and Sontheimer, 2009). The miRNAs mainly act to down-regulate cognate transcripts in a sequence specific manner. A single miRNA may regulate the expression of many target transcripts and thus acts as a probable master-switch in biological processes related to growth, development and response to environment (Djami-Tchatchou et al., 2017; Sharma et al., 2017). In plants, a miRNA gene is transcribed by RNA polymerase II into a primary miRNA transcript which is processed by DCL1 (DICER like 1) protein into a hairpin intermediate, called precursor miRNA (pre-miRNA). This is further acted upon by DCL1 to generate 20-24 nt long miRNA/miRNA^∗^ duplex in the nucleus. The duplex is then transported to cytoplasm where it is incorporated into the RISC (RNA inducing silencing complex) to guide the AGO (argonaute) protein which results in mRNA cleavage or translational repression (Bartel, 2004; Beauclair et al., 2010). The biogenesis and function of miRNA has been described and reviewed in many articles (Yang and Li, 2012; Tripathi et al., 2015; Mittal et al., 2016). Increasingly, the role of miRNAs is turning out to be much more complex than predicted earlier.

The advent of next generation sequencing (NGS) technologies along with computational approaches have revolutionized the identification and prediction of miRNAs and their targets (Motameny et al., 2010). The experimental validations and related studies have detailed the criteria for miRNA classification and this has formed the basis for several computational algorithms to screen NGS data sets (Unamba et al., 2015; Axtell and Meyers, 2018). Thus NGS is actively replacing hybridization-based methods to catalog and quantify miRNAs in a comprehensive and precise manner (Tripathi et al., 2015). The NGS data sets have also been useful in unraveling the transcriptome profiles thereby providing information on miRNA targets. Our early experiments reported the identification of tissue-preferential (Mittal et al., 2013) and stress-induced (Tripathi et al., 2015, 2018; Sharma et al., 2015; Goswami et al., 2017; Khan et al., 2018) expression patterns of rice miRNAs.

As on date around 10 rice miRNA database are available (**Supplementary Table S1**). The primary resources for rice miRNAs include miRBase^1^ (Griffiths-Jones, 2004; Griffiths-Jones et al., 2006, 2008) and Plant microRNA Database (PMRD^2^) (Zhang et al., 2009). In addition NCBI GEO/SRA has more than 30 experimental series comprising of hundreds of samples profiled for miRNA using high throughput sequencing approach. The miRBase contains 592 precursors and 713 mature rice miRNAs in the latest release (Version 21) (Kozomara and Griffiths-Jones, 2014) while PMRD contains 2773 miRNAs containing both experimentally validated and computationally predicted sequences (Zhang et al., 2010). PMRD also contains the promoter and miRNA target information for rice miRNAs. Studies based on miRNA sequences and that of their validated targets have facilitated identification of specific binding principles that led to the development of specific target prediction algorithms including psRNATarget^3^, PicTar (Krek et al., 2005), TargetScan (Lewis et al., 2003) and their combinations. These programs, however, are prone to report a large number of false positive targets. A number of related tools are also available for predicting the biological function of miRNAs and their targets genes like rice gene expression database (ROAD) (Cao et al., 2012), experimentally validated target gene expression database (PMTED^4^) (Sun et al., 2013), plant miRNA expression atlas database and web applications (PmiRExAT^5^) (Gurjar et al., 2016), rice expression profile database (RiceXPro^6^) (Sato et al., 2010), plant miRNA target identification tool (Target-align^7^ (Xie and Zhang, 2010), web server for the prediction of plant miRNA targets (TAPIR^8^) (Bonnet et al., 2010). These and similar databases provide the information relating to specific aspects only, for one or more plants. ARMOUR was designed to provide a cohesive database for complete analysis related to miRNAs in different rice varieties, that is not available so far.

The study of miRNA guided regulatory networks can spread a new light on genetic enhancements of stress tolerance in plants. Undoubtedly NGS has proven to be vital in detecting known and novel miRNAs and measuring their expression changes in rice (Fahlgren et al., 2007; Yang et al., 2011). The accumulation of this data has generated the need for a comprehensive and integrated database of miRNA:mRNA expression profile information and target information, implemented with biologist friendly user interface (UI) or user experience (UX). We present ARMOUR database that consolidates extensive datasets of miRNA deep sequencing studies in different rice varieties under various experimental conditions. It’s UX is designed with four different ways to interact with the database to examine the miRNA:mRNA interaction, with readily accessible information on expression levels. It provides a single platform for integrating information relating to genomic location, expression profiles, biological features, gene ontology and KEGG association for rice miRNA and their targets.

## Materials and Methods

### Database Design

**FIGURE 1:**
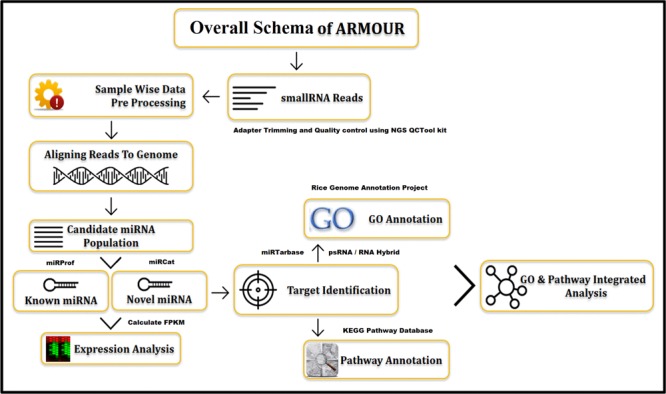
Overall schema of database development and functionality representation.

**FIGURE 2:**
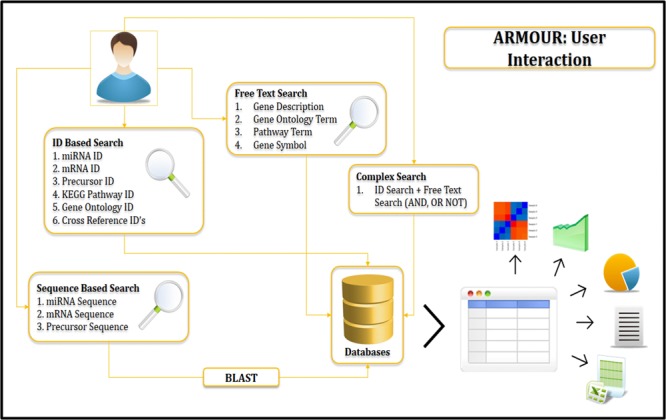
Database design and user interaction.

The UI was designed using HTML5 (Hyper Text Markup Language) and CSS (Cascading Style Sheets). All data for ARMOUR was stored in MySQL v5.6 database, which is a Relational Database Management System (RDBMS) and the most preferred choice for biological databases. In ARMOUR the UX is powered by Hypertext Preprocessor (PHP) and jQuery, while SQL queries were optimized for memory efficient data retrieval and specialized scripts were used for hassle free database updating. The application programming interface (API) is unique feature of the database design and schema (**Figure 1**). ER (Entity Relationship) tool was used to create an ER diagram that places a total of 13 tables representing comprehensive and heterogeneous information related to miRNA and mRNA that are seamlessly connected in ARMOUR. The UI or UX was designed in four different ways to interact with the database to examine the miRNA:mRNA interaction with expression level information readily accessible (**Figure 2**). ARMOUR database is designed to be both potable (multiple applications can be developed on the database) and scalable (additional data can be updated time to time) without changing the design.

### Database Content

The database includes miRNAs identified from seven varieties of rice under 38 different developmental and stressed conditions (**Table 1**) representing leaf, root, flag-leaf, and panicle tissues of seven varieties of rice grown under normal, salt stress and heat stress conditions. The cultivars used are traditional Pusa Basmati 1, dwarf Annapurna, high yielding Lalat, dry season Satabdi, heat-susceptible BPT 5206, salt-tolerant Pokkali and drought/heat-tolerant Nagina 22. The known or identified rice miRNAs were retrieved from miRBase Rel 21 and searched in the NGS libraries while the novel putative miRNAs were predicted using miRcat tool in sRNA Workbench from rice genes or transcripts (Stocks et al., 2012). The database includes 689 known and 1664 predicted novel miRNAs (**Table 2**). All miRNAs may not be necessarily present in each of the libraries, however, their large numbers indicate the abundance and variability of miRNAs.

**Table 1:** Libraries details.

Cultivar	Tissue	Physiological condition	Character
**Pokkali**	Leaf	Salt stress and normal	Salt tolerant
**Pusa Basmati**	Flag leaf, panicle, leaf, root, flower	Salt stress, heat stress, and normal	Susceptible
**Lalat**	Flag leaf, panicle	heat stress and normal	Susceptible
**Shatabdi**	Panicle	heat stress and normal	Susceptible
**Annapurna**	Panicle	heat stress and normal	Heat tolerant
**BPT-5206**	Flag leaf, panicle	heat stress and normal	Susceptible
**Nagina-22**	Flag leaf, panicle	heat stress and normal	Drought tolerant

**Table 2:** Key database statistics.

Feature	Number
**Unique experimental conditions covered**	38
**Known miRNAs**	689
**Predicted novel miRNAs**	1664
**miRNA targets covered**	14890
**Unique miRNA: mRNA relationships**	26321
**Database cross references**	8
**Pathways covered**	118
**Gene ontology – biological processes**	2400
**Gene ontology – molecular function**	1868
**Gene ontology – cellular component**	487

### Nomenclature and Gene Annotation

Annotation data type was restricted to VARCHAR to ensure simplicity, fast query and retrieval while all images were generated on the fly through server side scripting. The nomenclature for known miRNA and their target transcripts, in ARMOUR, complies to the standards of miRBase and Rice Genome Annotation Project nomenclature (Kawahara et al., 2013). miRNA identifiers include miRBase ID, miRBase Accession and miRNA Family. All the predicted miRNAs are represented as “Novel-*n*” (where *n* is an integer ranging from 1 to 1664) designated as miRNA ID. The predicted novel miRNA ID will be updated periodically as they are validated and entered into miRBase. Using miRNA ID, the miRNA and the precursor sequences can be fetched out, information on the target transcripts can be obtained and the expression profiles of the miRNAs can be obtained. The gene or transcript identifiers that are covered include Entrez gene ID, MSU7 transcript ID, RAPDB gene ID, Uniprot ID, and RefSEQ mRNA ID. The gene or transcript annotation includes gene description, RNA type (coding or non-coding), chromosome number, gene start, gene end, orientation, transcript length, gene ontology (GO) and Kyoto Encyclopedia of Genes and Genomes (KEGG) pathway information (Kanehisa and Goto, 2000).

### Expression Data and Fold Change Analysis

To study the accurate expression profiles of miRNA, the read counts were normalized and provided as log2 of reads per million (RPM; fold expression) in the ARMOUR database. Data normalization was done to ensure minimal redundancy levels since miRNA:mRNA relationship is one is to many and vice versa. Fold Change (FC) analysis can be performed by selecting a reference sample or condition followed by comparison against one or more samples or conditions. The formula used for FC is same as used earlier (Campbell et al., 2015).

$$\begin{array}{c} \mathrm{Fold}\;\mathrm{expression}\;=\;log2\;\left(\mathrm{RPM}\right)\\ \mathrm{FC}\;=\;log2\;\left(\mathrm{RPM}\right)\;\mathrm{test}\;\left(-\right)\;log2\;\left(\mathrm{RPM}\right)\;\mathrm{control} \end{array}$$

### miRNA Targets Identification and Scoring

Transcripts targeted by both known and predicted miRNAs were identified using psRNATarget: A Plant Small RNA Target Analysis Server (Dai and Zhao, 2011) using the rice transcript dataset from Rice Annotation Project Version 7. Targets were qualified based on satisfying all of the following criteria (a) Expectation scores between 0 to 3 (Zhang, 2005), (b) Unpaired Energy Score (UPE) ranging from 0.0 to 25.0, (c) High scoring Segment Pair (HSP) size of 15 to 20 nucleotides, (d) Maximum number of transcripts targeted less than or equal to 200 hits, (e) Flanking length around target for accessibility analysis ranging from 17 nucleotides upstream and 13 nucleotides downstream around target size (Kertesz et al., 2007), and (f) central mismatch leading to translational inhibition centered between 9 to 11 nucleotide from the 5′ end of the miRNA (Brodersen et al., 2008). The information pertaining to transcripts targeted by the miRNA includes type of inhibition (cleavage or translation repression) and multiplicity (number of sites in the transcript likely to be targeted by the miRNA). Later the targets were also analyzed using PsRNA Target ver 2017. However, the 2017 version algorithm gave almost five times more target per miRNAs (∼30–40 mRNA per miRNA) in comparison to 2011 version due to the modified algorithm, indicating the possibility of including false positives (comparison results for 10 miRNAs is provided in **Supplementary Table S2**). So it was taken care to incorporate the targets that were common to both predictions.

The identified target transcripts were further annotated by identifying their GO categories, that include well planned terminologies, which describe the biological process (P), molecular function (F), and cellular components (C) of gene products, obtained from Rice Genome Annotation Project^9^. To find the associated pathways which are being targeted and affected by these miRNAs through their target transcripts, the information on associated KEGG pathway annotations to the selected target transcripts are also mentioned.

## Results and Discussion

### Expression Data and Analysis

**FIGURE 3:**
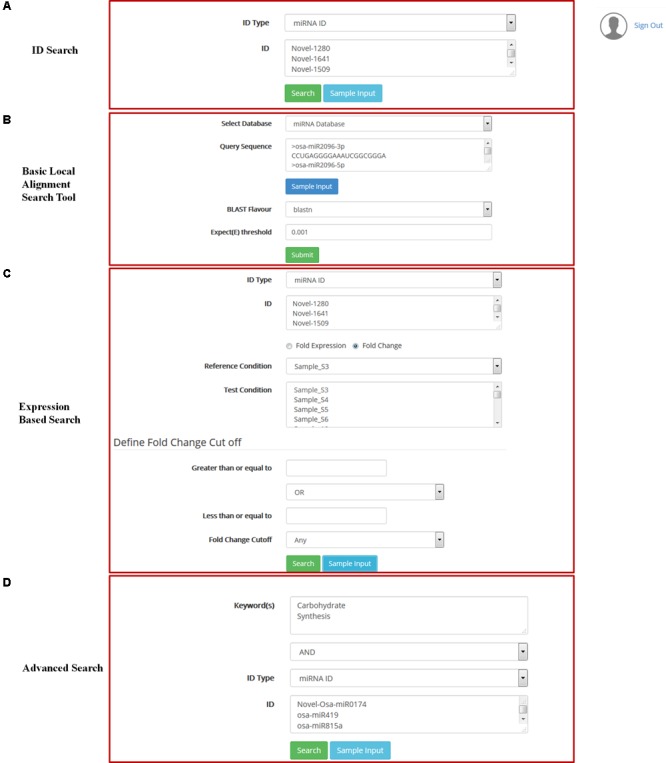
Accessing of DATA: **(A)** Searching of data using Ids, **(B)** searching of data using sequence, **(C)** Expression based search, **(D)** Advance search.

The behavior of the known and predicted miRNAs listed in the database can be inferred from their expression status in the various libraries representing response to different environmental conditions in different rice varieties. miRNA expression analysis can be performed by filtering the results based on the cut off for fold expression using conditional filter, either in one sample or condition or in multiple samples or conditions. Fold expression can be easily calculated and the results are provided as a flexible Fold Change (FC) cut off to identify up and down regulated miRNA. Fold expression and FC analysis results are provided in an interactive table with expression values and annotation (**Figure 3**). Within the database there is a provision of using the miRNAs (provided as examples) for retrieving and comparing their expression patterns. The results can be downloaded and used for presentation in many ways (Sharma et al., 2015; Goswami et al., 2017; Khan et al., 2018; Tripathi et al., 2018).

ARMOUR will not just act as a knowledge reservoir but also an analytic tool for the researchers to analyze and compare their data with the existing data. The users can send a request to add their own experimental data and compare with the available experimental datasets, thereby allowing sharing and comparision of data between research groups working on rice.

### Access to the Database

ARMOUR home page provides an interactive view to the user to become familiar with the database. Key aspects of UI include responsive front end (Device independent), floating frames, fluid design for tables and reader friendly color coding. Various aspects like pre-loaded sample identifiers for each query interface, client side validation of queries, simplified BLAST search, keyword filter on the query results, sorting functionality on table headers, interactive matrix table representation in advanced search, summary charts for GO and pathway queries, sequence level highlighting of miRNA structure and floating buttons ensure ease of navigation on all screens. The user can access any of these search options to retrieve all information related to the query. The results are reported in a unique correlation matrix format, which provides all the information related to the query. The matrix is populated with the number of gene hits that can be re-queried into the database.

#### Sequence Based Search

Local installation of NCBI-BLAST v 2.2.3 (Johnson et al., 2008) is implemented in ARMOUR database. Backend databases available for search comprise of miRNA precursor sequence database and miRNA mature sequence database from miRBase as well as transcript database obtained from MSU rice database release 7 (Ouyang et al., 2007). BLAST variants that are available for users include blastn, tblastn and tblastx with user defined expect threshold (*E*-Value) cut-off option for searching (**Figure 3**). The search can be performed directly from the home page by using the miRNA IDs and by other given keys to obtain information on the miRNA, precursor sequences, expression profiles and target transcripts.

### Query Builder Interface

ARMOUR has a unique query builder interface for the users that allows the database to be queried using unlimited keywords or in combination with a list of miRNA or transcript identifiers. Keywords can be searched on Gene description, GO and KEGG pathways and results are reported in a unique correlation matrix format. The matrix is populated with the number of gene hits that can be re-queried into the database. The matrix also shows how many genes are unique and being shared within and across multiple key words (**Figure 3**).

**FIGURE 4:**
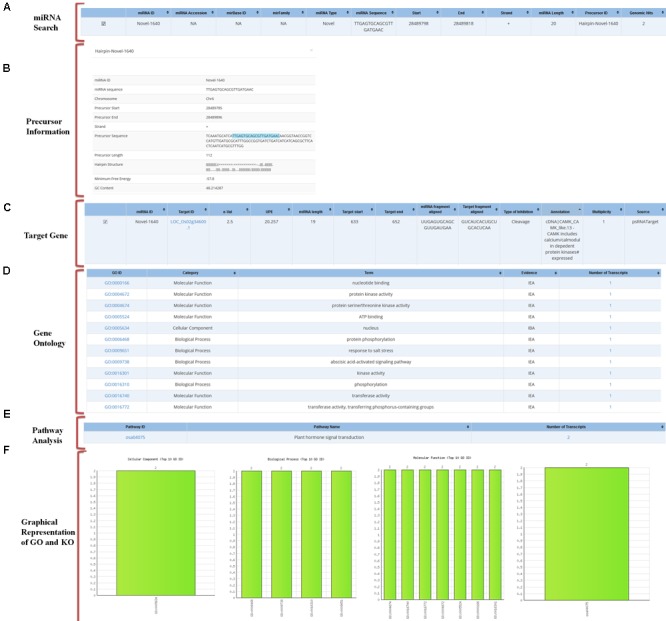
Overall representation of a sample miRNA with an example of “novel-1640” miRNA. **(A)** Result of miRNA search for the selected miRNA IDs. **(B)** Precursor sequence information. **(C)** Predicted target gene information. **(D)** Gene ontology of the predicted target. **(E)** Pathway analysis of the predicted target. **(F)** Graphical representation of the target’s Gene ontology.

Using an ID of any miRNA the user can analyze all related data to the query in the database. The GO output includes information on the details of GO category to which selected targets belong and the number of the selected targets belonging to the particular GO. To find the associated pathways which are being targeted and affected by these miRNAs through their target transcripts, the information on associated KEGG pathway annotations to the selected target transcripts are also mentioned (**Figure 4**).

## Conclusion and Perspectives

Rice (*Oryza sativa*) is one of the most important crops in the world, with a well mapped and well characterized genome. There are several publications relating to role of rice miRNAs in plant development and response to stress (among others Sanan-Mishra et al., 2009; Lv et al., 2010; Ding et al., 2011; Atwell et al., 2014; Mittal et al., 2016). miRNAs are small, non-protein-coding RNA molecules that in association with specific protein complexes, regulate the expression of specific gene products in a sequence-specific manner (Wahid et al., 2010). Number of computational algorithms or tools are available that allow fast and confident prediction of miRNAs and their targets (Jones-Rhoades et al., 2006). The miRNAs have been implicated in numerous developmental and disease states, but their function in distinct biological pathways and phenotypes remains largely unknown (Bazzini et al., 2007; Sharma et al., 2017). Hence, a comprehensive integrome resource of rice miRNA and mRNA would fasten the meta-analysis of miRNA mediated gene regulation in rice at various conditions. This entailed the development of, a rice miRNA and mRNA integrated analysis and user-friendly resource, ARMOUR. It serves as a huge reservoir of known and new rice miRNAs and their expression atlas.

The database is unique in its specific design as it integrates miRNA expression data, from different tissues and varieties of rice, with the predicted target information. This enables analyzing phenotypes and biological functions by associating gene level information from conventional canonical pathways and GO. It provides users with multiple interfaces to interact with the backend database through gene expression, sequence based search and custom query builder. Therefore, it provides a useful resource for researchers investigating the miRNAs and their functional impacts in rice or related cereal crops.

Current version of ARMOUR is focused only on sRNA sequencing for miRNA expression analysis. The existing data in the current version of ARMOUR will be further enriched by adding more experimental data. As future extensions we intend to include and use RNA-seq data and the PARE data or degradome analysis from same tissues and rice varieties for identification and expression analysis of targets. This will give a complete picture and an easy way to compare the expression of miRNAs and their corresponding targets. We envision that the users will also share and integrate their experimental data into ARMOUR to formulate it into a collaborative platform to access and share compiled experimentally validated data resource exclusively for studying rice miRNA and its interaction with its target mRNA. ARMOUR will be constantly updated as soon as new data is available.

## Availability and Requirements

Project name: ARMOUR – a rice miRNA: mRNA interaction resource

Project home pages: http://armour.icgeb.trieste.it/login and https://www.icgeb.org/armour.html

Operating system(s): Platform independent

Programming languages: HTML, CSS, MySQL, jQuery.SQL

License: Not required. Each new user can register with his/her details using a valid e-mail ID

Any restrictions to use by non-academics: None.

## Author Contributions

NS-M: conceived and designed the experiments. AT, KG, and RS: analyzed the data. RS, MV, HG, and NS-M: contributed in designing of database and analysis tools. NS-M, AT, KG, and MV: wrote the paper.

## Conflict of Interest Statement

The authors declare that the research was conducted in the absence of any commercial or financial relationships that could be construed as a potential conflict of interest.

## Footnotes

1 http://www.mirbase.org/

2 http://bioinformatics.cau.edu.cn/PMRD/

3 http://plantgrn.noble.org/psRNATarget

4 http://pmted.agrinome.org/index.jsp

5 http://pmirexat.nabi.res.in

6 http://ricexpro.dna.affrc.go.jp/index.html

7 http://www.leonxie.com/targetAlign.php

8 http://bioinformatics.psb.ugent.be/webtools/tapir/

9 http://rice.plantbiology.msu.edu/annotation_pseudo_goslim.shtml

## Supplementary Material

TABLE S1 List of rice miRNA database.

TABLE S2 Target predictions results for 10 miRNAs as determined using psRNA Target versions 2011 and 2017, respectively.

## Abbreviations

ARMOUR: A rice miRNA: mRNA interaction resource
miRNA: microRNA
